# Experimental and Numerical Studies of Gas Permeability through Orthogonal Networks for Isotropic Porous Material

**DOI:** 10.3390/ma14143832

**Published:** 2021-07-08

**Authors:** Grzegorz Wałowski

**Affiliations:** 1 Institute of Technology and Life Sciences—National Research Institute Falenty, 3 Hrabska Avenue, 05-090 Raszyn, Poland; g.walowski@itp.edu.pl; 2 Branch Poznan, Department of Renewable Energies, 67 Biskupińska Street, 60-463 Poznań, Poland

**Keywords:** isotropic porous material, polyamide sinter, orthogonal networks, gas permeability, CFD, turbulence

## Abstract

With regard to the problem of gas flow through isotropic porous deposits, the issues were considered in the category of description of gas movement mechanisms for structural models of the skeleton. As part of experimental tests of gas permeability through porous material in the form of polyamide, the numerical simulation method was used, using the *k*–*ε* turbulence model. The analysis of hydrodynamic phenomena occurring in the porous material made it possible to confront experimental research with numerical calculations. The analysis shows that, for a porous polyamide bed, there is a certain limit range of gas velocity (10^−4^–1) ms^−1^ at which flow resistance is the lowest. On the other hand, the highest value of the flow resistance is gradually achieved in the range of gas velocity (1–10) ms^−1^. This is due to the different structure of the isotropic polyamide material. The validation of the numerical model with experimental data indicates the validity of the adopted research methodology. It was found that the permeability characteristics of the tested porous material practically did not depend on the direction of gas flow. For porous polyamide, the permeability characteristic is non-linear, which, from the point of view of the measurements carried out, indicates the advantage of turbulent gas flow over its laminar movement. The novelty of the article is a proprietary method of measuring gas permeability for a cube-shaped sample made of a material constituting a sinter of spherical particles of equal dimensions. The method enables the determination of gas flow (in each flow direction) in microchannels forming an orthogonal network, characteristic of isotropic materials.

## 1. Introduction

A significant variety of porous deposits, both for their use in the industrial technology and their presence in the environment, makes the fluid flow through these types of materials extremely complex and ultimately unrecognized. At the same time, the literature on the subject discusses this issue in different ways, as there is more emphasis on the application of fluid flow hydrodynamics through porous (granular or frame-structured) beds than on basic research. Each porous medium has a specific porosity, and its flow structure is subject to this porosity and to dimensions (diameter) and shapes of its channels at a given length of the channel. Another specific feature of porous bodies is their capability of storing and transporting liquids under the action of external and internal powers. Aksielrud and Altszuler [1] argue that the gas flow through porous media whose channels (pores) have several millimeters or fewer is dominated by some process phenomena that emerge from the hydrodynamics of viscous liquid flow, whereas in case of the flows through structures whose pores are very small (tenths of micrometer), those phenomena are hampered by physical–chemical and diffusive mechanical effects that occur at the boundary of the phases. This fact is also proven by other authors [2,3], but the diversity between those phenomena dissipates when gas keeps moving highly intensively.

As for the hydrodynamic approach, the mechanism of gas flow in porous deposits is strictly connected with a geometrical structure of the porous medium and is subject to dimensions (diameter), shapes and tortuosity of the channels [4].

For the flow in the free space between the grains, it is assumed that the entire space resulting from a given porosity is an active space with open channels interconnected with each other. The additional complexity of hydrodynamics results from the fact that these structures are compact (rigid) deposits, which cannot be relaxed in any way during the pressure increase in the system. Consequently, the flow resistance during flowing around the winding spaces will be distinguished by isotropic channels.

To determine the porosity system of a homogeneous shape, the capillary models that are isotropic channels [5,6,7,8,9,10,11,12,13,14] should be mentioned. The model solutions formulated by researchers [11,13,15,16] relate to the shape of interconnections between capillaries and are based on the following assumptions. Circular/symmetrical capillary models of uniform shape are characterized by the following:

- Cylindrical capillary tube with constant radius [11],
- Two interconnected capillaries of different radii [13],
- Circular conical shape [15],
- Hyperbolic longitudinal section [16].

In most cases, the first group of models is characterized by the simplest geometry of pores, i.e., cylindrically shaped flow channels according to Burdine’s model [11]. The base element of this model only comprises a cylindrical capillary with a constant radius. Burdine’s model can be successfully used in heat and mass transfer process calculations [17,18,19,20]. On the other hand, the capillary model formulated by Mualem [13] describes the configuration of the porous deposit by using two mutually combined capillaries with different radii. In this model, the cross-section ratio of capillaries is constant for the entire deposit. Then Michaels [15] developed Mualem’s model, assuming that the capillary comprises a set of cylinders connected in series and with two different diameters. These cylinders with large and small diameters are connected alternately. On the other hand, a much more complex model of capillary with a periodically changed cross-section was proposed by Petersen [16]. This model comprises a symmetrical and circular area that results from the rotation of symmetrical sections of the hyperbole around its own axis. The individual sections of such capillary (segments with greater and smaller diameters) are connected alternatively. This model significantly better, compared to Michaels’ one, reflects the structure of the real capillary as in this case the change to the diameter of individual segments of capillaries is smooth, not jumpy [17]. An extension of the group of uniformly shaped circular and symmetrical capillaries is structural (double) models. The liquid flows through the symmetrical porous matrix comprising a set of circular and symmetrical capillaries. It has the same radius and length (as Burdin’s model) and a set of capillaries according to Michael’s or Petersen’s models [15,16]. Aksielrud and Altszuler [1] add that the studies [21,22,23,24] have analysed Towbin’s uniform capillary model [1] with a shape of the spherical deposit. In this case, however, this model was too general as a great number of bodies has a capillary/porous structure that results from the combined spherical grains with small diameters.

The mechanisms of gas transport that dominate in a specific geometry of pores and in specific thermodynamic conditions are discussed by Rose [25]. This author classifies the movement of liquid in the deposit according to the following streams:

(a) Sub-capillary stream when the movement is forced under pressure and is carried out at a higher temperature;
(b) Capillary stream that is observed when capillary forces and the surface tension occur;
(c) Super-capillary stream that emerges under the action of gravity forces and described by general laws of hydraulics.

On the other hand, Gertis and Werner [26] classify means of gas transport in porous structures into the following:

(a) Laminar flow that is observed when molecules collide with one another within the extent of the capillaries’ radii;
(b) Diffusion that occurs as an automatic process of mixing gas particles until their composition is fully completed;
(c) Knudsen transport, *Kn*, defined as a number of collisions of a particle with walls of pores with respect to a total number of collisions among particles [27].

The article reviews selected models of porous structure and describes the issues of the mechanism of gas flow through porous isotropic structures.

As part of the work, experimental studies of gas flow hydrodynamics through isotropic porous material in the form of polyamide were performed—a capillary/porous model of a homogeneous system made of spherical grains of small diameter connected together. The measurements were carried out on a specially prepared test stand. An experimental method for determining the hydrodynamic parameters constituting the permeability coefficient and its numerical verification for determining the nature of gas flow was presented. In comparison with the cited literature, the use of the developed gas permeability model was indicated, thanks to which the flow characteristics can be universally presented in terms of gas flow mechanisms. Numerical simulation was used for a material of the same shape. Simulation programs contain Computational Fluid Dynamics (CFD) codes and are a useful tool used when gas flows through porous materials. Carrying out numerical simulation allows for a detailed analysis of hydrodynamic phenomena taking place. In the CFD modeling, the Kolmogorov hypothesis was used for the transport of kinetic energy of turbulence, *k*, and the transport of the rate of dissipation of kinetic turbulence energy, *ε*.

The aim of the study was to develop the concept of a numerical model of an isotropic porous bed, built of sintered balls of the same diameter, for which it is easy to determine the values of permeability and porosity. The idea of a numerical isotropic model of a porous bed is based on experimental studies carried out for a cube-shaped sample. Referring in this way to the research material (sintered polyamide), it has been shown that the gas movement for a porous bed is described by the Navier–Stokes equations, and in the case of turbulent flow by the Reynolds equation. The boundary values for the proper within a porous bed are velocity and pressure distribution values obtained from the solution of the Navier–Stokes equation or the Reynolds equation.

It was undertaken to develop a gas permeability model (knowing the flow rate, pressure drop, material porosity and type of gas), because the literature has not found an example to determine the gas permeability coefficient in a universal way. The permeability (hydrodynamic parameter) was considered in this category, describing the mechanisms of gas movement in porous structures for the development of hydrodynamics in materials of a new generation of clean energy sources [28,29,30].

## 2. Materials and Method

This research material—porous sinter (cube 20 mm × 20 mm × 20 mm)—directly comes from the polymerization of spherical grained powders constituting the solid part of the porous material, i.e., a skeletal structure (manufacturer: “INWET” S.A.) and is used as the so-called gas distributor designed for aeration of liquid systems. Other names are porous polyethylene or porous polyamide, depending on the type of plastics used.

The apparent density, *ϑ_poz_*, of the porous material was determined by measuring the total volume of the *V_pr_* sample (by immersing it into water) and its mass, which, in this case, corresponds to the mass of the solid body *m_pr_* = *m_st_*. Equation (1) is as follows:

(1) $$\vartheta_{poz}=\frac{m_{st}}{V_{pr}}.\;$$

The absolute porosity of the porous material, *Ψ_b_*, is lculated from the quotient of the free volume, *V_swo_*, and the total volume of the sample, *V_pr._* After some significant transformations, this definition may look like the following (2):

(2) $$\Psi_{b}=\frac{V_{swo}}{V_{pr}}=1-\frac{V_{st}}{V_{pr}}=1-\left(\frac{m_{st}}{\vartheta_{st}}\right)\left(\frac{\vartheta_{poz}}{m_{st}}\right)=1-\frac{\vartheta_{poz}}{\vartheta_{st}}.\;$$

The *ψ* porosity coefficient was determined, which on the other hand characterizes the degree of density of the porous medium [31]. Definition (3) is as follows:

(3) $$\psi =\frac{\Psi_{b}}{1-\Psi_{b}}.\;$$

The parameters determined for the tested materials (samples) are set forth in Table 1 together with sample numbers and average values of those parameters.

### 2.1. Experimental Procedure

To achieve the research goal, detailed experimental studies were carried out to evaluate the gas permeability of the porous material.

**Figure 1:**
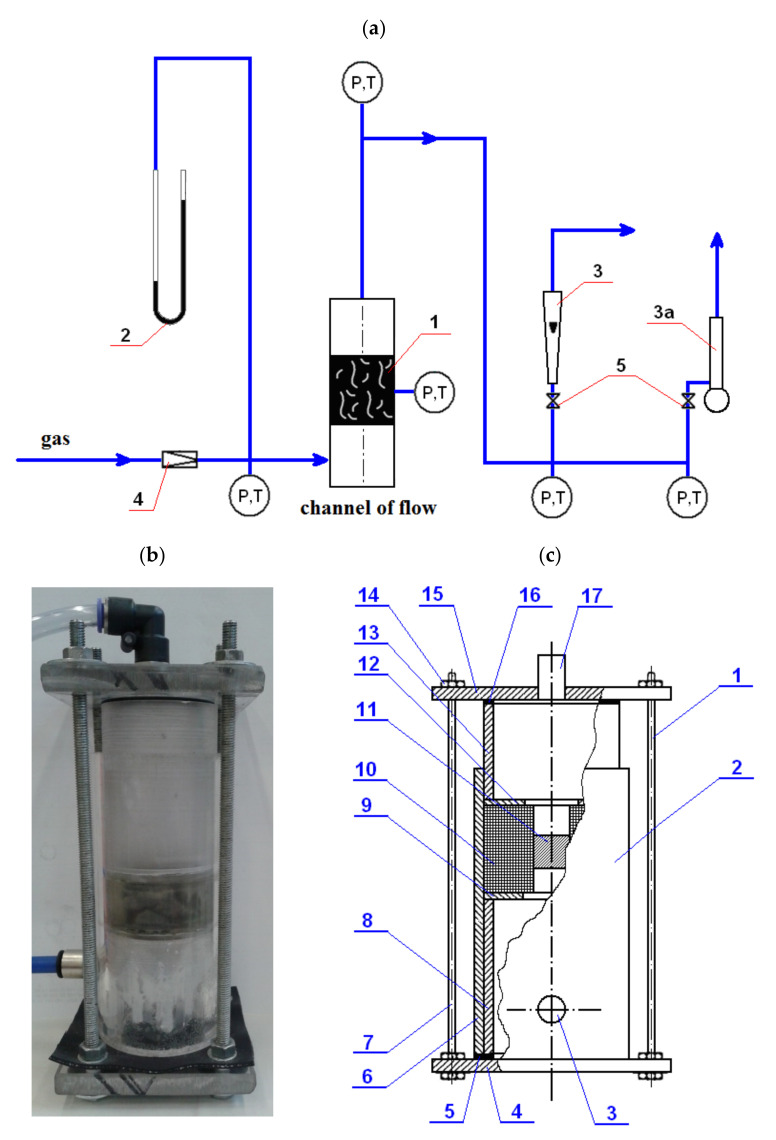
Measurement system for testing the permeability of porous materials for directed gas flow. (**a**) Diagram: 1—porous material (sample), 2—differential pressure manometer, 3—rotameter (3a—bubble flowmeter), 4—pressure reducers and 5—control valve; P = manometer and T = thermometer. (**b**) Gas permeability meter view. (**c**) Construction diagram: 1—casing, 2—cylindrical body, 3—gas inlet pipe, 4—bottom base, 5—bottom seal, 6—bottom fixed outer part, 7—threaded rod, 8—bottom fixed inner part, 9—lower thrust ring, 10—measuring cell, 11—porous material sample, 12—upper thrust sample, 13—upper movable sleeve, 14—nut, 15—upper base, 16—upper gasket and 17—gas outlet pipe.

Figure 1a shows the measuring system for testing the permeability of tests carried out on cubic-shaped samples. In this system, the gas flow was always directed in relation to any chosen fixed axis, i.e., X, Y or Z. In each case, the gas flowed through the sample in the slow pressure regime, the reference pressure in the reduction valve was (0.04–0.16) MPa and the gas freely was flying out. The air was taken from a compressed air system with a reference temperature of 18 °C. The pressure drop in a particular measurement system was measured with differential manometers installed in the flow measurement system at the gas inlet to the material sample. The gas flow was measured with the use of flotation rotameters calibrated with a bellows gas meter before the tests were carried out. All gas stream measurements were compared to normal conditions (293 K, 1013 hPa). Air process values measured during the experiment were gas viscosity 0.000018 Pas, gas density (1.258–2.684) kg/m^3^, ambient temperature and pressure (4.7–128.7) kPa.

The measurement system, or more precisely the construction of the permeability meter [32] used in the directed gas flow along the axis of the selected sample X, Y and Z, used for the samples of a cubic shape, is shown in Figure 1b,c.

This permeability meter [32] is designed for testing the permeability of samples in the form of a cubic solid in any direction of its axis, which is possible by applying a special sealing system of the measuring cell that enables measuring the permeability towards any direction X, Y, Z of the location of the measurement sample in the measurement system according to the scheme as shown in Figure 1a.

**Figure 2:**
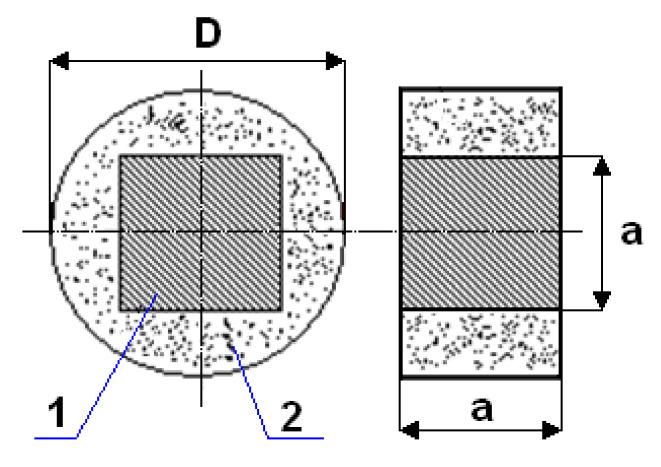
Diagram of the measuring cell: 1—porous material; 2—sealing material (cubic sample); D = 49 mm; a = 20 mm.

The scheme of this measurement cell together with the marking of the sealing material and the measurement sample is shown in Figure 2.

In order to determine the gas permeability coefficient for the sample, first the system (Figure 1a) is set up. Then the measuring cell (Figure 2) with the previously cutout of the porous material bed, the cube-shaped sample with dimensions 20 × 20 × 20 mm, is placed in the gas permeability meter (Figure 1c) on the ring the lower 9 of the fixed lower part of the inner 8 of the body 2 and presses the upper ring 12 of the movable upper part 13 of the body 2 by means of the base 15 of the upper casing 1 and threaded rods 7 with nuts 14. Air is passed through the sample 11 so positioned according to the test method. Air of known density, at ambient temperature, is supplied through the inlet to the pressure reducer under the preset pressure in the range (0.4–1.6) 10^5^ MPa. The volumetric flow of the gas flowing through the sample is measured in the given flow direction X. At the same time, the pressure value is read on the pressure gauge. In this way, two process parameters are measured, namely air flow and pressure, which are the resistance to the sample of the porous material. The air from the flow meter and the cap is discharged through the outlet to the environment (Figure 1a). Then, after disassembling the permeability meter, the sample itself is rotated by an angle of 90° and placed back in the measuring cell (Figure 2). After the permeability meter is assembled, the procedure of measuring the same two is repeated process parameters by passing air through the sample along successive flow directions Y and Z. Knowing the volumetric flow, air density and flow resistance of air flowing through the sample for each flow direction, the gas permeability coefficient is determined.

### 2.2. Numerical Research

As a principle, theoretical considerations with regard to the gas flow through porous media are based on the models of flow through axial microchannels, capillaries by application of the laws pertaining to flows, including Poiseuille’s law, as well as the indications of the pressure drop applying simplifying assumptions. However, in each case, the models are based on the quantities describing the physical characteristics of the porous medium, such as shape of pores, their size and arrangement, and bed permeability.

The movement of viscous fluid described by Navier–Stokes (N–S) is a complete system of dependencies, on the basis of which it is possible to determine, inter alia, pressure and velocity field of such a fluid [33]. Depending on the particular needs and the type of the investigated problem, e.g., incompressible and compressible fluid, there are different ways in which the averaging of this system of equations is performed. Most commonly this objective applies equations averaged over time or space, suitably to the method based on the Reynolds hypothesis, as for instance based on the *k*–*ε* model or small-scale approximation methods suited to model Large Eddy Simulation (LES). The averaging of the velocity profile in time leads to the derivation of Reynolds equations expressing the law of the conservation of momentum in averaged turbulent flow [34].

On the Kolmogorov scale, *λ*, in description of the N–S, the dominant role is attributed to the term derived from viscosity. The gas motion occurs in the laminar conditions and it is characterized by a considerable level of energy dissipation, which is converted into heat. On the vorticity scale *l*, the gas motion occurs in the conditions where the term representing the viscosity in description the N–S is close to being disregarded. This, in turn, leads to motion of gas driven by inertia, as the energy of the motion is extracted from the spatially extensive areas: it passes through a cascade of vortices without its dissipation in the areas with the smaller capacity. For larger scales in space—on the channel scale—there are coherent structures in the form of vortices, which partly synchronize their pace of motion. On this scale, forces which induce motion occur and this motion is driven by means of large-scale structures.

Under the assumption that a fluid has homogenous and incompressible properties *ρ* = const., *η* = const., averaging over time gives this leads to the Reynolds hypothesis this leads to the description which relates the mean values of the components of the velocity vector. The Reynolds stress tensor results in additional difficulties in the solution of the models based on such averaging Reynolds Averaged Navier–Stokes equations (RANS), because it adds new unknowns and now becomes unsealed. This requires the use of additional solutions that bind the components of the tensor. A compromise in these circumstances can be sought in the application of the *k*–*ε* model. It was first proposed by Chou [35], and then it was further modified on multiple occasions [36]; hence, it is currently one of the most commonly used turbulence model with regard to incompressible fluids with small velocities. The Reynolds stress tensor for turbulent flow is defined by an additional equation in this model (4):

(4) $$\tau_{ij}^{*}=\eta_{T}\left({\bar{u}}_{i,j}+{\bar{u}}_{j,i}\right)-\frac{2}{3}\bar{\rho }k\delta_{ij},$$

for

(5) $$\eta_{T}=\rho \;c_{\mu }\;k^{2}/\varepsilon$$

Despite the fact that the *k*–*ε* model is strictly dedicated to the incompressible flow, this model is successfully used in the area of gas flow, in particular in conditions where the solutions for the influence of local disturbance on the hydrodynamic parameter are tested for fluid flow. The concept of this model uses the closure of the N–S described by two additional differential equations for the kinetic energy transfer of turbulence *k* and the turbulence transport of the kinetic energy dissipation rate *ε*, which can be estimated using the Kolmogorov hypothesis [37]. This hypothesis indicates a relationship between macroscopic flow structures expressed by a linear flow scale taking into account dissipation, which characterizes even the smallest scales of vortices.

It can be seen that another model from the RANS group, namely the *k*–*ω* model, is also a model for the kinetic energy transport of turbulence *k*, which in this case is related to the vorticity *ω*. In this case, the system consists of two equations. However, its application for various types of flows does not provide satisfactory solutions due to the assumption of the scalar nature of vortex viscosity [38].

Regardless of the method used, numerical calculations must include the derivation of a geometric representation of the modeled object. In ANSYS Fluent, this is performed in the Design Modeler module [39]. The numerical network should faithfully reproduce the entire area occupied by the gas, ensuring an appropriate ratio of microchannel shape fragmentation to its volume, so that it can be treated as a single computing cell. For this purpose, it is necessary to look for a compromise between the level of detail and the number of nodes in the numerical network, which affects the computation time. Generating the network consists in the discretization of the model space, i.e., describing how it can be divided into finite elements that define the so-called control volume. Each element of the larger whole expressed by geometry creates a space in which the gas flow is balanced according to the equations determining the conservation of mass, momentum and energy. The literature on the subject [40] emphasizes that the numerical network is one of the most important aspects of model construction, because its density determines the precision of calculations and the time needed to determine the solution. This network should also assume the highest density in areas where exchange processes should take place, such as the occurrence of turbulent motion and sudden change of flow directions. A dedicated module called Mesh in ANSYS CFX Fluent is used to generate the network. This software is used to prepare data and obtain numerical solutions to complex problems in the field of gas transportation thanks to the Solver Preference module in Fluent. The accuracy of the results depends basically on the appropriate selection of the method—in this case, it is the Insert method. For this procedure, it is valuable to select Sweep calculation, as it affects the structure of the mesh elements when generating the mesh with respect to the microchannel. The standard *k*–*ε* model requires much smaller discretization networks as it uses the time-averaged N–S description. The averaging time of the velocity field leads to the statement of the Reynolds equation assuming the following (6):

(6) $$Re=\frac{ud\rho }{\eta }.\;$$

In this case, the number of nodal points on the appropriate spatial lattice should be proportional to *Re*^9/4^, which results from the statistical theory of the turbulent motion [34,41].

## 3. Results and Discussion

Darcy’s law is still the basis for a detailed analysis of fluid flow in porous media. In its original form, this law describes the permeability conditions of various types of granular beds by referring to the filtration mechanism during the laminar flow of water through a layer of sand, which is the standard granular medium. Taking into account the variability of the properties of the liquid, the velocity through the porous bed will be proportional to the change in density *ρ* and inversely proportional to the change in viscosity *η* [42]. Then the Darcy equation describing the permeability *Q* of the porous bed takes the following Formula (7):

(7) $$Q=KA_{o}\frac{\rho g}{\eta }\frac{\Delta h}{L}$$

This formula remains one of the features of the modern description of this phenomenon, although it only applies to laminar flow. The *K* factor in Equation (7) describes the so-called the permeability of a porous medium, and its value, as shown by the Darcy model, is characteristic of a given porous medium. Since this coefficient (by definition) has a surface dimension, its value from a hydrodynamic point of view—as a characteristic dimension—is very often regarded as a certain geometrical feature characterizing the overall permeability of the porous material. On the other hand, the value of this permeability depends not only on the filtration properties of the porous medium (its structure, particle size, their density, porosity, etc.), but also on the physical properties of the fluid, especially its viscosity [43]. As a rule, this factor does not depend on the shape and size of the bed itself. Of course, the Darcy model is also applicable to the description of pressure flows. Then, for Equation (7), one can obtain the following (8):

(8) $$Q=KA_{o}\frac{\Delta P}{\eta L}\to K=\eta \frac{Q}{A_{o}}\frac{L}{\Delta P}$$

The last equation shows that for a given volume flow *Q*, the permeability of a porous bed can be determined experimentally if the fluid properties *η* and the geometric parameters of the flow system *A_o_* are known. The pressure drop *ΔP* across the bed is therefore an experimental value. If the hydrodynamic parameters are known (flow rate, pressure drop, porosity of the material and, of course, the type of gas), the value of the permeability coefficient can be determined experimentally. Then Relation (9) can be written as follows:

(9) $$K_{V}=\frac{Q_{g}}{\sqrt{\frac{\Delta P_{zm}}{\rho_{g}}}}$$

### 3.1. Experimental Assessment

A reference point to the assessment of fluid dynamics of gas flow through a porous material was a porous polyamide comprising the agglomerate of spherical particles with identical dimensions, about 0.1 mm diameter. This material has the absolute porosity of 32% Table 1. To obtain the research objective, the detailed experimental tests were conducted to assess the gas permeability of skeletal material porous—for one sample depending on the flow direction X, Y and Z and the averaged direction XYZ—by using air as a working medium Table 2.

**Figure 3:**
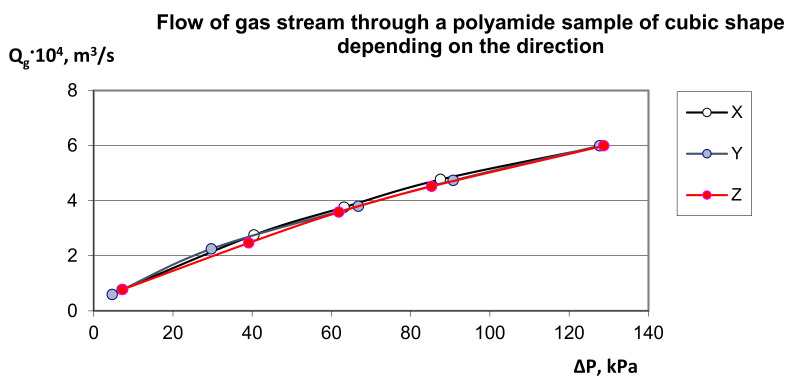
Distribution of experimental points characterizing the air flow *Q_g_* by depositing a cube-shaped sample of polyamide with dimensions of 20 mm × 20 mm × 20 mm, with reference to for the measured flow resistance *ΔP* depending on the direction of flow, i.e., X, Y and Z.

**Figure 4:**
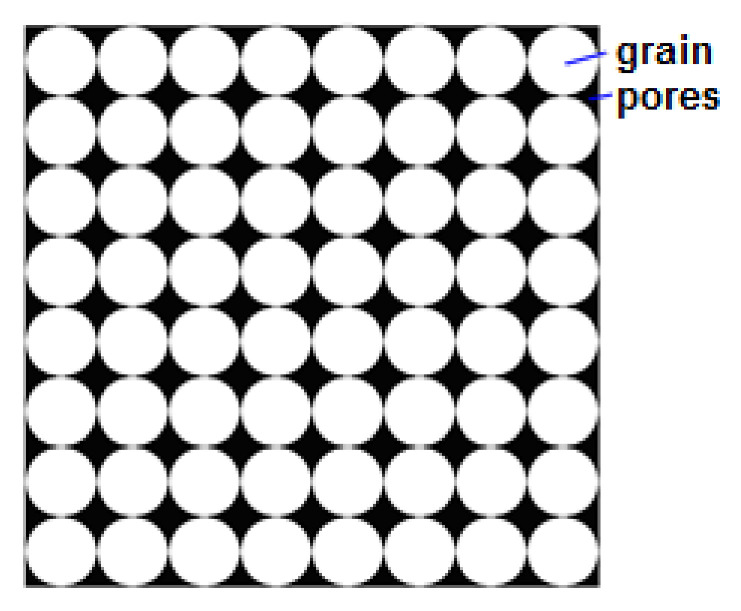
Research material—porous sinter: example of a porous material structure (black = free space; white = frame-based structure).

By assumption, the own model (9) was compared to an alternative approach to the assessment of pressure drop resulting from local resistances in the porous structure to the multiplanar flow directions X, Y and Z (Figure 3). The distribution of experimental points presented in Figure 3 proves that the permeability characteristics of this material practically do not depend on the direction of gas flow, which proves the symmetrical structure of its structure Figure 4, which is similar to a diamond pattern and has isotropic properties. Interestingly, for porous polyamide the permeability characteristic is non-linear (Figure 3), which, in the measuring range, indicates the advantage of turbulent gas flow over its laminar flow.

The assessment of gas permeability through porous beds is important both for process and technological reasons. In both cases, numerous attempts are made to find effective methods of predicting permeability of porous materials, as well as effective methods of measuring and verifying the methods of this assessment.

For example, in the mathematical model of decarbonation of a ball-shaped sample made of Hills calcium carbonate [44], it assumes that carbon dioxide flow follows Knudsen’s diffusion principles [45,46]. Consequently, carbon dioxide transport equations use Knudsen diffusion coefficients. Khinast et al. Assume similarly in the paper [47], regarding limestone decarbonation. Still another approach is presented in [44]. An effective carbon dioxide transport factor through the layer of quicklime is introduced in the description of carbon dioxide transport during limestone decarbonation. On the other hand, in [48,49], the Darcy equation [50,51] is used to describe the displacement of the thermal dissociation reaction front of limestone, in which the specific permeability of the solid reaction product characterizes its texture [52] and determines the amount of carbon dioxide flow.

Thus, there is a relationship between the porosity of the bed and the type of flows that can occur in it [53]. A characteristic feature of Poiseuille flow is the viscous flow resistance, which determines the gas flow velocity in the capillaries forming the porous medium. In Knudsen diffusion flow, the frequency of collisions of gas particles with capillary walls is higher than the frequency of intermolecular collisions. Gas loses momentum due to collisions with capillary walls. Volmer flow occurring as a result of surface diffusion is particularly important in limestone decarbonation. According to Bretsznajder [54], carbon dioxide forms a two-dimensional gas on the surface formed by calcium oxide, which moves from places with low carbon dioxide adsorption potential to places with higher carbon dioxide adsorption potential, favoring the course of the reverse reaction to the thermal dissociation reaction of calcium carbonate. At large times, for which *Kn* < 10^−2^, there is a Poiseuille flow. When the average free path of gas molecules in relation to the diameter of the capillary (pores) through which the gas flows is in the range *Kn* (10^−2^–10), the flow in the capillary (time) occurs under the influence of its own partial pressure gradient. Surface diffusion of two-dimensional gas occurs for the value of Knudsen number *Kn* > 10^−2^.

According to the principles adopted in chemical technology [55], the description of limestone decarbonation uses, among others, the mass balance equation of transported carbon dioxide through the resulting quick lime layer and the heat balance equation recorded for the reaction front [44,47,48,49,56].

Another example is the Skotniczny study [57] for a porous medium, which was a bed formed of glass beads (ballotin). According to Skotniczny, for the fluid flow through the isotropic medium, the value of the Reynolds *Re_d_* number is important, which is a characteristic dimension for the diameter of the solid phase particle. The author of the study noted that for flows with *Re_d_* > 1 there are deviations from Darcy’s law due to the increasing share of an additional factor, which is the quadratic resistance of the flow. This phenomenon, interpreted by Forchheimer [58], indicates the extension of the basic Darcy equation by a term describing the effect of quadratic resistance on pressure drop in a porous bed.

The empirical observations [59,60,61,62,63,64,65,66] indicate that the movement of air and other gases taking place at a distance not very far from the rigid impermeable walls is most often of a turbulent flow nature [60]. The final effect of the internal transport of kinetic turbulence energy is its dissipation occurring due to fluid viscosity, in the range of the finest vortex structures characterizing the flow [59]. Turbulent flow is an unstable strongly vortex motion, for which the occurrence of vortices of various size scales is characteristic. Large-scale vortices are carriers of the greatest kinetic energy, but they do not dissipate it; instead, they transfer it to smaller-scale vortices. As a result, the energy dissipates finely in vortices of the smallest scale and converts it into heat [61,62,63]. Turbulence may or may not develop when the Reynolds number Re exceeds the critical value *Re_cr_* [64]. Detailed experimental studies have shown that this value is not universal, but depends on the specific type of fluid movement. For example, for flow through straight round tubes *Re_cr_* = 2300. Due to the complex nature of turbulent movement, its universally accepted definition has not yet existed, and all existing terms are fragmentary and incomplete. According to the classic Hinze definition [62], turbulent flow is an irregular fluid movement in which velocity and other flow parameters experience unpredictable, random changes in space and time (so-called fluctuation). However, certain average values characterizing the flow may be determined.

The cause of turbulent traffic for many years was an unsolved problem, among others due to the incompatibility of this phenomenon with the variational principles of mechanics. As Reynolds [64] suggested, and Lin ultimately showed [65,66,67], the cause of turbulence is fluid instability, in which, in addition to the regular behavior characteristic of laminar flow, there is a continuous spectrum of alternative behavior. Because none of them are highlighted in any particular way, the actual fluid motion process occurs randomly from one alternative behavior to another. This manifests itself in the form of a chaotic turbulent movement. The role of viscosity in creating turbulence is clearly ambivalent. An increase in viscosity stabilizes the nature of fluid movement. On the other hand, viscosity is an important cause of instability, and ideal fluid flows, in which the viscosity of the definition equals zero, are always regular and no turbulence occurs. According to the Reynolds idea, the vector of fluid velocity in turbulent motion can be presented as the sum of the average value and random fluctuation. Consequently, non-zero component values for the Reynolds tensor are finally obtained for the movement of real fluids. Depending on how the Reynolds tensor is represented, the appropriate turbulence model is obtained.

The reference books also describe statistical–physical models describing the course of the liquid flow process in porous structures, as illustrated in the studies [2,3,19,68,69]. The fluid movement has a different character for turbulent flow in the free stream and in the pore space. The effect of the occurrence of a non-zero speed at the media border, called the slip velocity, is difficult to accurately describe due to the significant differences in the equations of motion describing the flow of fluid in a free stream and in a porous bed.

From the phenomenological point of view, the flow of a liquid through a porous medium may be subject to various hydrodynamic criteria, which are influenced by the structure of the medium, the type of fluid (single or multiphase) and the method of forcing the flow (gravity, pressure). A wide range of carefully analyzed publications, incl. in the works [70,71,72,73,74,75] describing this issue on the scientific and research grounds, it concerns the filtration process and is generally equated with the laminar flow of a fluid through granular beds according to Darcy’s law [76]. It does not exhaust many other examples of fluid flow through porous media. For turbulent fluid flow, the Forchheimer [77] and Szczelkaczew [78] models should be distinguished. A more advanced description of the flow through the spatial system of capillaries in the form of meandering channels is also found in Brinkhman [79]. The literature on the subject discusses other models of hydrodynamics of single and multiphase fluids flowing through porous media, taking into account the influence of fluid properties and the type of porous medium on the flow through granular beds [80,81,82,83,84,85,86,87]. A separate group of the practical use of the gas permeability methods is studies on the quality assessment of construction materials, particularly concretes [88,89,90,91,92]. Research upon the gas permeability of concretes also enables indirectly assessing the capillary/porous structure of concretes which results from their composition of components, including a kind of the cement applied. This research is exemplified by the experiments carried out by Tracz [92]. In this research the author determines the permeability of concretes for the nitrogen flow and by using Darcy’s law. The results of these tests indicate a clear influence of the total porosity of the mortar isolated from concrete on its permeability. Another method for measuring the concrete gas permeability is Torrent’s method which is, among others, described in the study by Śliwiński and Tracz [91]. This method is restricted to the sample of concrete with a thickness of several centimeters to which a vacuum suction pipe is connected with a special measuring head. The permeability measurement consisted of creating the vacuum of 10–50 mbar on the controlled surface of the tested element; then, after obtaining the required vacuum and deactivating the vacuum pump, the pressure freely equalizes to the atmospheric pressure. It was determined that the time and intensity of pressure equalization depended on the concrete permeability, which proves its porosity and structural construction. In addition, concretes are characterized by very small gas permeability (10^−4^ mDa) which is proved by numerous research, including those carried out by Glinicki [88], the results of which also cover the concrete quality classification criteria. The thesis that identifies the flow nature (laminar or turbulent) with the permeability of porous materials contributed to numerous experimental research, in particular with respect to the gas flow conditions in such structures [93,94,95,96].

The methods used to measure gas permeability through porous beds are very diverse in the literature and it can be assumed that the only common feature of these methods is the construction of samplers, although there are no uniformly standardized methods for this assessment. An additional difficulty in this respect is the fact that the samples used in the research have a different form and shape, and most often they are model beds, which do not always correspond to real conditions. A slightly different aspect is that the gas permeability assessment is generally performed in one selected flow direction of the prepared sample, which, in relation to porous natural materials, leads to large quantitative errors. This state of affairs does not facilitate the transfer of measurement results to real conditions, nor does it facilitate the establishment of clear criteria for scale transfer. This results in the individualization of methods for assessing gas permeability through porous beds, which are usually based on experimental formulas. The theoretical assumptions resulting from the interpretation of the hydrodynamics of the gas flow through various media are formulated with greatly diversified models (mathematical and experimental), considering a straight/axial flow (Poiseuille’s laminar flow model) or a more complex filtration process (the Darcy model that is the only possible one for the laminar movement) or numerous modifications of those models for specific structural conditions of the deposit, based on the experimental criteria of the fluid movement in the closed spaces. The literature-based modifications most frequently refer to the determination of flow resistances, albeit they are dedicated for granular media or for their specific forms such as the infill of column apparatuses.

Referring to the analysed methods for measuring the gas flow in porous structures and methods for assessing the gas permeability through porous deposits, it must be stated that a great difficulty for the application of the literature-based models and their adaptation to other (compared to their assumptions) process conditions is a result of a very diversified structure of porous materials, particularly the shape of pores, their cross-section, mutual connections that enable the liquid flow or the porosity size whose relatively high value does not always mean the greater efficiency of the frame-structured porous materials.

**Figure 5:**
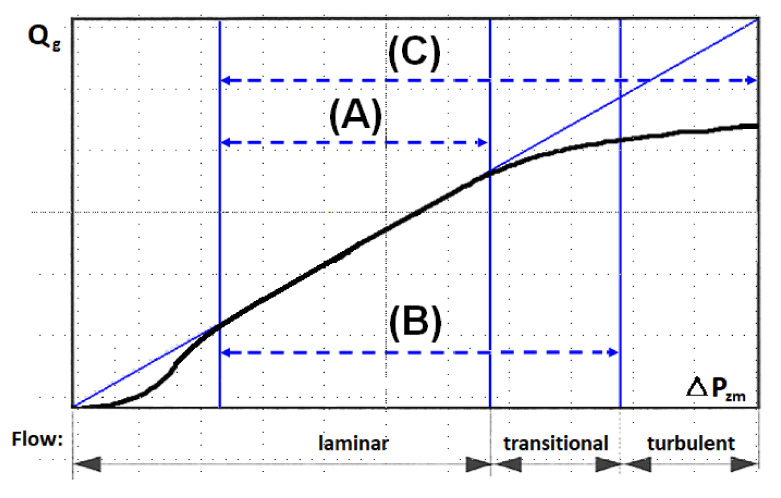
Gas flow as a function of gas flow resistance in a granular medium (author’s own elaboration) range: A—Darcy, B—Forchheimer, C—Brinkman.

Generally, it may be assumed that the non-linear dependence (Figure 3) between the pressure decrease and the liquid flow is characteristic for a great amount of inertia forces that are significantly higher than the impact of viscosity forces—Forchheimer’s model [77]. In its general approach, the model described by Forchheimer is also called the Darcy–Forchheimer model, delineates a decrease in pressure in the porous deposit with respect to viscosity forces characteristic for the laminar flow (Darcy law) and to inertia forces that consider the significance of the turbulent flow. The characteristic Forchheimer coefficient, for which the permeability parameter *K*, is determined experimentally, but there are also known calculation formulas that depend the value of this coefficient on the deposit porosity and the channel tortuosity. A slightly broader definition of the Forchheimer model is provided by Brinkman who observed that elementary liquid flows moving at various speeds would affect one another with additional viscosity forces, depending on the flow of wall effects with diversified sections [97]; this is confirmed by a fully developed turbulent flow [98,99,100,101]. By comparing the Darcy, Forchheimer and Brinkman models, it is possible to compare the scope of their application as shown in Figure 5. The characteristics of the permeability function with respect to the pressure decrease in the deposit show that except for the Darcy range (A) in which the linear dependence of this function applies, in the remaining ranges depicting the transitional and turbulent flows (B and C), this function is non-linear. When in the laminar area the liquid flow is affected by diffusion phenomena, then in this area the permeability description is also non-linear. Generally, the greater flow turbulence gives rise to the greater impact of inertia forces on the generation of flow resistances.

In all the cases described in the literature there is no uniform view of the possibility of using in the hydrodynamics description the criteria for flow pores or the assessment of gas permeability (flow stream). In addition, there are considerable variations in the approach to the experimental assessment of the permeability parameters. It hinders to a great extent the possibility of the research results, which, consequently, leads to difficulties in adapting the existing computational models. An additional problem is the proper assessment of the nature of the flow and the actual flow parameters resulting from the structure of the porous deposit.

**Figure 6:**
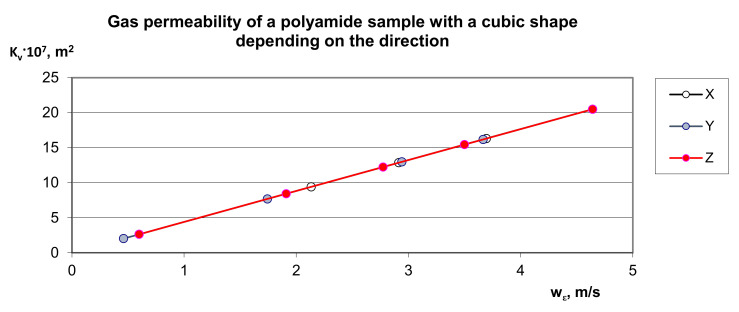
Distribution of experimental points characterizing the *K_V_* gas permeability coefficient through a 20 mm × 20 mm × 20 mm cubic polyamide bed with respect to the measured gas flow velocity, *w_ε_*, depending on the flow direction X, Y and Z.

A significant variety of porous deposits, both for their use in the industrial technology and their presence in the environment, makes the flow of liquids through this kind of materials tremendously complex and not finally recognized. The reference books referring to this issue put a greater emphasis on the application-based recognition of hydrodynamics of the liquid flow through porous deposits (granular or skeletal) compared to the fundamental research; therefore, the use of the gas permeability model was proposed. Figure 6 shows the gas permeability characteristics related to the apparent velocity in the deposit depending on the direction of gas flow, i.e., X, Y and Z. The observed phenomenon of gas permeability indicates the correct interpretation of the proposed model (9), because regardless of the direction of gas flow in the characteristic channels forming orthogonal networks, the curves overlap.

**Figure 7:**
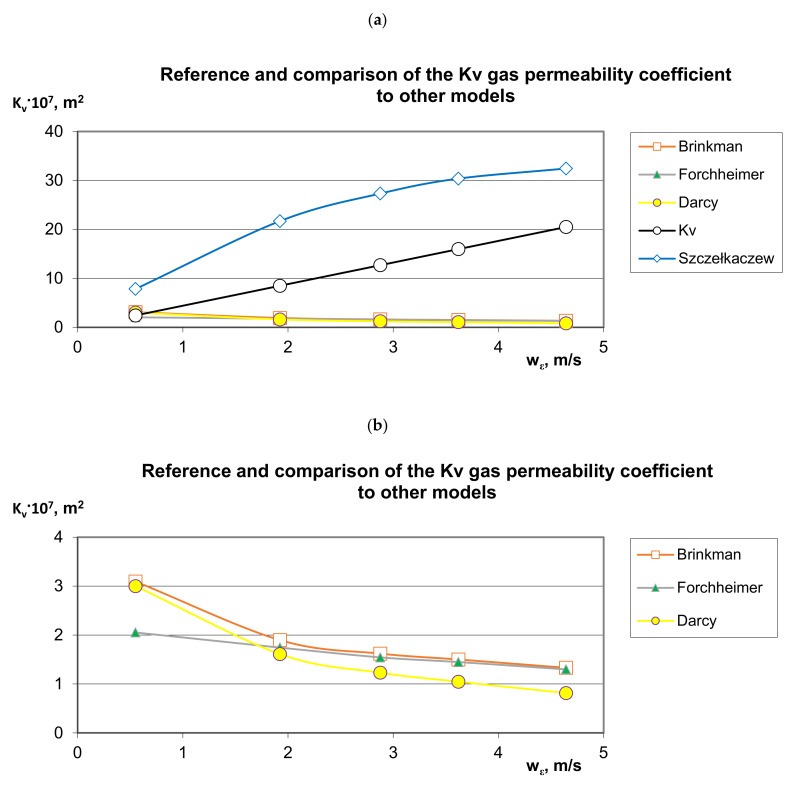
Reference and comparison of the *K_v_* gas permeability coefficient to other models—distribution of experimental points characterizing the gas permeability of a 20 mm × 20 mm × 20 mm cubic polyamide bed with respect to the measured gas flow velocity depending on the averaged direction of flow XYZ: (**a**) general chart and (**b**) fragment of the chart.

The developed model (9) was related to the Brinkman, Forchheimer, Darcy and Szczełkaczew models (Figure 7); it can be noticed that the author’s own model universally fits into the previously unrecognized area and fills the gap for the research niche. The author’s own model also takes into account the mean volumetric value calculated as a weighted mean for the generalized XYZ direction (Figure 7). It can be seen in Figure 7 that very different results are obtained in terms of the applied calculation method. There are three characteristic tendencies of changes in the value of the gas permeability coefficient with the increase in apparent velocity: increasing (Figure 7a), proportionally increasing (Figure 7a) and decreasing (presented in detail in Figure 7b). According to the Darcy, Forchheimer and Brinkman models, the downward trend indicates a greater throttling effect than the gas velocity is available for. A completely different character of the changes in the gas permeability coefficient is described by the Szczełkaczew model; this indicates an increase in the permeability intensity with an increase in gas velocity, eliminating the effects of throttling. The gas flow intensity may indicate the full free surface with a regular structure (Figure 4). Taking into account such large discrepancies and numerous limitations in the use of models known from the literature in terms of the structure of porous materials and the approach to the assessment of the hydrodynamics of gas flow through porous materials—based on the author’s own model—it was observed that the gas permeability coefficient increases proportionally to the apparent velocity.

This demonstrates the limitations of the use of other authors’ models in relation to the proprietary model, which is universal and has no limits to its application.

### 3.2. Numerical Assessment

**Figure 8:**
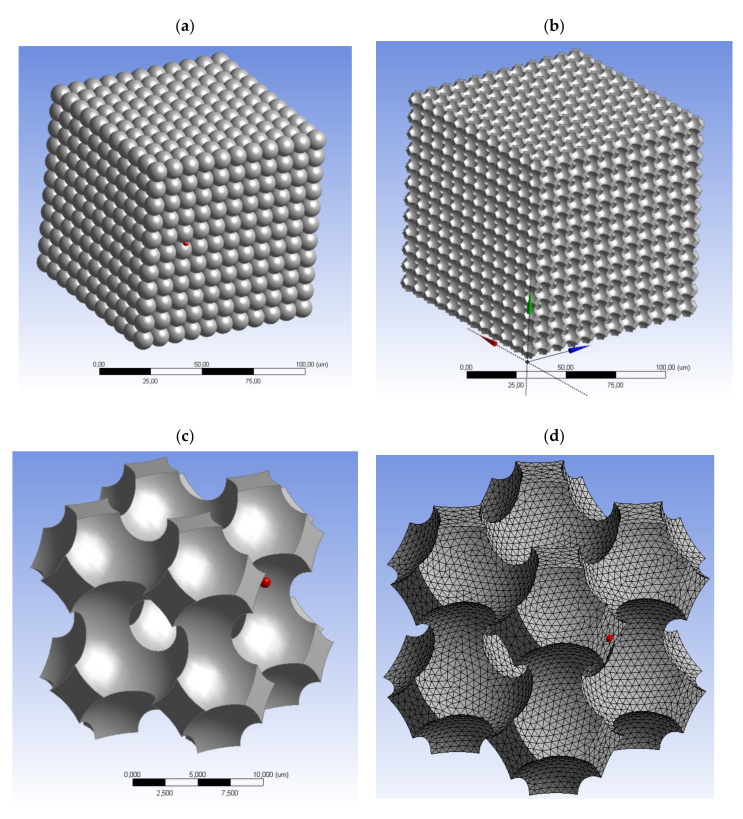
Geometrical model: (**a**) porous bed with a symmetric cube structure *a* = 20 mm, *d_k_* = 10 μm, *Ψ* = 32.2%, elementary bed model structure; (**b**) structure of symmetric porous spaces; (**c**) elementary unit of cellular open for flow structure; (**d**) calculation network 85∙10^3^ cells.

The characteristic type of polyamide material is shown in Figure 8, which, in its structure, is representative of the microchannels forming the orthogonal network that is typical for polyamide sinter. The network model of the geometry of gas flow forms the structural equivalent to these materials, as it represents the structure and porosity of the materials in question. The porous sinter (polyamide) demonstrating the characteristics of isotropy Figure 3 forms in this respect a practical model of the porous material whose equivalent number for a sample volume with the cubic shape is equal to *n* = 1 (Table 1). In addition, a porous medium characterized by a porous structure Figure 8a is repeatable in terms of its layout of all its components, which leads to the conclusion about repetitive geometry of the calculation network along its entire volume. On the basis of the model of the geometry of porous spaces (Figure 8b), a specific gas flow unit was identified to establish the elementary model of its geometry (Figure 8c). This was followed by its discretization for different mesh densities were used in the calculations, as shown in the examples in Figure 8d.

**Figure 9:**
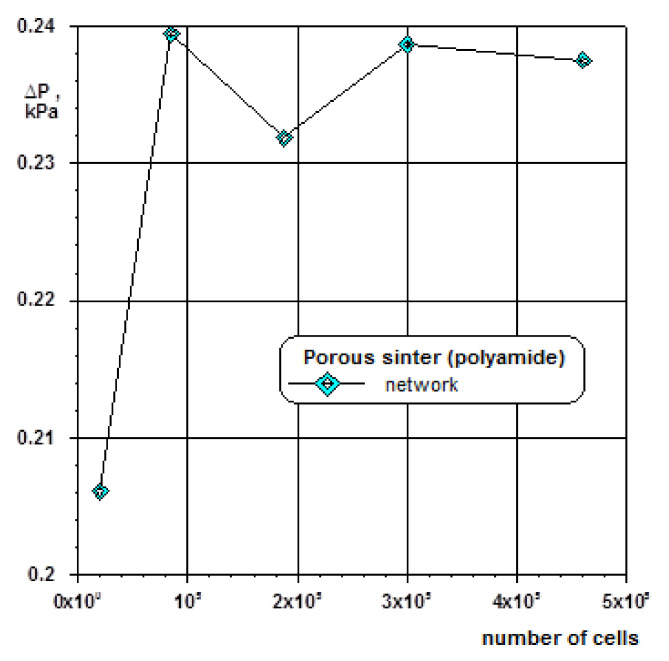
Discretization of model of geometry for cube-shaped network; *a* = 20 mm, *d_k_* = 10 μm, *Ψ* = 32.2%.

From the assessment of the impact of the number of grid cells on pressure distribution in the orthogonal network it follows that the repeatable results of calculation are obtained for 85∙10^3^ and more number of cells (Figure 9).

**Figure 10:**
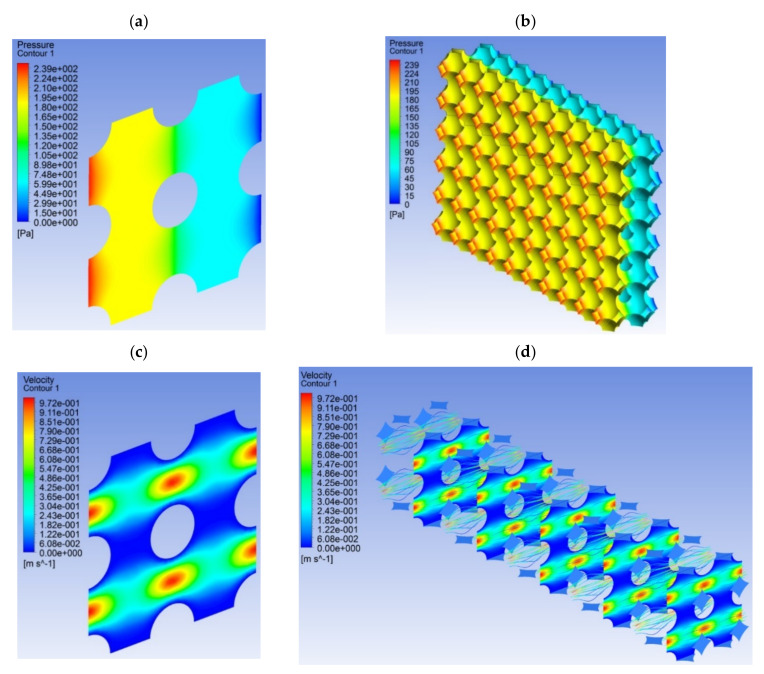
Distribution of gas flow in the framework structure for the cross-section of (**a**) the elementary unit’s pressure, Pa; (**b**) times multiple of the elementary unit’s pressure, Pa; (**c**) the elementary unit’s velocity, ms^-1^; (**d**) times multiple of the elementary unit’s velocity, ms^−1^.

In order to analyze the fluid dynamics of gas flow through microchannels in a porous material, there must be a step related to the determination of the material properties, determination of environmental conditions and selection of boundary conditions. This stage is one of the most important aspects of using the CFD method, as the range of initial and boundary conditions significantly affects the quality of the obtained numerical solution. The analyzed issue concerned the definition of the superficial criteria needed to define the boundary conditions at the inlet: pressure-inlet and velocity-inlet. For this purpose, selected areas were identified along the model object, which were later called zones. Such zones are taken into account in the calculation procedure as a representation of the limits for which selected input quantities, both measurable and observable, are determined. The inlet boundary condition was defined as the air velocity at the inlet to the duct with the value *u_g_* = 0.195 ms^–1^. The boundary condition for the outlet pressure was assumed as determined by the static pressure. During the simulation, the input parameters were set as they were during the experimental tests. For example, the property of a gas under temperature and pressure conditions was as follows: gas viscosity 0.000018 Pas, gas density (1.258–2.684) kg/m^3^, ambient temperature and pressure (4.7–128.7) kPa. In numerical research, only an example of the structure formed by the real sample is presented. The focus was on relating the hydrodynamic conditions in the numerical object. The calculation results characterizing the pressure change during gas flow in a cubic structural system are shown in Figure 10a. For a multiplied volume sample, analogous calculation results are shown in Figure 10b. The same calculation criteria were used to describe the velocity field as shown in Figure 10c,d. In each of these cases, a consistent set of calculation results was obtained, which indicates a well-applied methodology for modeling the geometric structure of porous materials.

**Figure 11:**
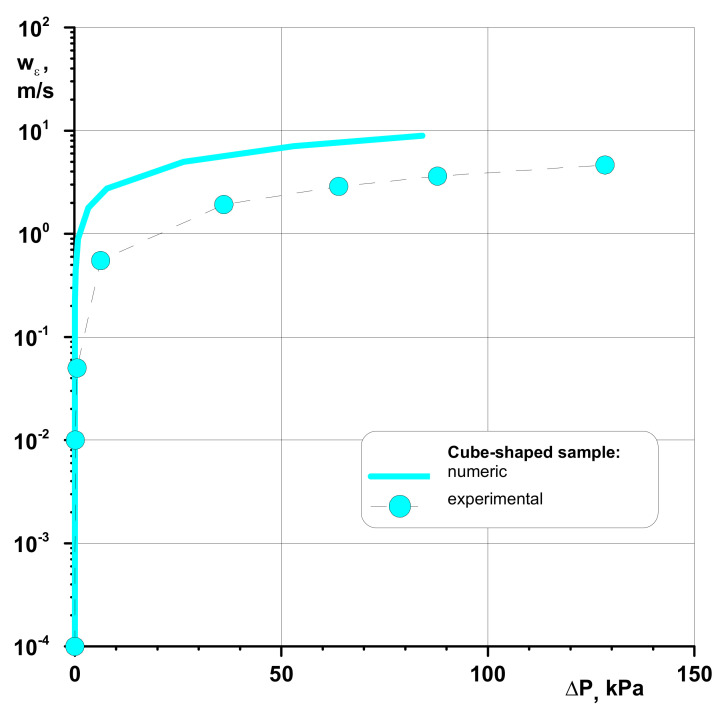
Summary of experimental and numerical results for porous sinter.

The summary of the results, yet referring to the comparison of the results of the numerical and experimental data, is presented in Figure 11. The results of the numerical studies—which are related to the mean structure of these materials—offer a suitable characteristic of the conditions resulting from the fluid dynamics of the gas flow through a porous bed. The tendency to note variations in the permeability function that was observed for a constant geometric network represented by the velocity of the gas flow offers a considerable degree of repeatability; it is does not present distinct results from the ones gained from the experiment. The lower results gained from the experiment for the case of polyamide (Figure 11) are associated with the imperfections of the structure of this material, formed by sintered material with a cube-shaped structure, as it comprises a large number of defects in the capillary flow channel, which result from the characteristics of the sintering process. This could lead to formation of an asymmetric structure [102,103], which was not accounted for in the numerical calculations. The analysis of the results presented in Figure 11 also leads to the statement that for a porous isotropic material, there must be a boundary range of gas velocities for which the pressure drop assumes the lowest value for around 1 ms^−1^ for polyamide. This distinction is associated with the diverse structure of these materials. The results confirm the well-accepted calculation methodology and make it possible to evaluate the dynamics of gas flows through porous structures. This study can form a considerable contribution to the validation of the results of testing performed in the conditions of actual porous beds.

## 4. Conclusions

Experimental studies have shown that the porous polyamide sinter is an isotropic material. The results of numerical simulations enabled the quantitative analysis of gas flow through the structure of orthogonal microchannels using the turbulent *k*–*ε* model taking into account the Kolmogorov hypothesis. The validation of the numerical model with experimental data indicates the validity of the adopted research methodology. The following conclusions can be drawn from this study:

(1) It was found that the permeability characteristics of the tested porous material practically did not depend on the direction of gas flow. This indicates a symmetrical structure and isotropic properties. For porous polyamide, the permeability characteristic is non-linear, which, from the point of view of the measurements carried out, indicates the advantage of turbulent gas flow over its laminar movement. The porous medium characterized by a spherical bed structure is reproducible in every element in terms of its structure.

(2) The results of numerical calculations concerning the structure of the porous material in the form of sintered polyamide are well characterized by the conditions resulting from gas flow through the porous bed. The tendency of changes in the permeability function is repeatable and does not differ much from the experimental values.

(3) The analysis of the presented results shows that for a porous polyamide bed there is a certain limit range of gas velocity (10^−4^−1) ms^−1^, at which the flow resistance is the lowest. On the other hand, the highest value of the flow resistance is gradually achieved in the range of gas velocity (1–10) ms^−1^. This is due to the different structure of the isotropic polyamide material.

(4) Practically in all cases (literature models) there is no uniform view on the possibility of using criteria characteristic for flow resistance or assessment of permeability (flow stream) in the description of hydrodynamics. In addition, the literature on the subject indicates large discrepancies in the approach to the experimental assessment of permeability parameters. This makes it difficult to compare research results, which in turn translates into difficulties in adapting the already existing computational models. Moreover, the problem is the correct assessment of the nature of the flow and flow parameters resulting from the structure of the porous bed.

(5) The innovation of the work consists in the concept of solving the above problems and a completely new approach to gas permeability based on the assessment of process phenomena in many technological aspects relating to the properties of materials—resulting from the hydrodynamics of gas flow, e.g., example by isotropic material.

(6) A novelty in the research is the use of a measuring system to test the permeability of a porous material for a directed gas flow. The use of a proprietary model of the gas permeability coefficient enables a universal approach to research on the hydrodynamics of porous deposits.

## Figures and Tables

**Table 1:** Characteristic of research material—sintered porous (polyamide) (author’s own study).

Porosity Absolute	Equivalent Pore Diameter	Indicator Porosity	Density:		Cubic Solid:	
			Apparent	Skeleton	Volume of the Sample	Equivalent Number of Volume *
*Ψ_b_*, %	*d_e_*, μm	*ψ*	*ϑ_poz_*, kg/m^3^	*ϑ_st_*, kg/m^3^	*V*, dm^3^	*n*
32.3	10.0	0.5	772	1140	0.008	1

* Times the volume of a cube with the dimensions of 20 mm × 20 mm × 20 mm in relation to the volume of a sample (body) of any shape.

**Table 2:** The results of gas permeability measurements depending on the flow direction air, 18 °C.

Research Material		Porous Sinter (Polyamide)	
Sample Number		XV-1	
No.	Reference pressure *P_o_*, MPa	Gas stream *V*∙10^3^, m^3^/s	Resistance flow *ΔP_exp_*, kPa
direction of gas flow: X			
1	0.04	0.078	7.2
2	0.08	0.276	40.4
3	0.1	0.376	63.2
4	0.12	0.477	87.5
5	0.16	0.600	128.6
direction of gas flow: Y			
1	0.04	0.060	4.7
2	0.08	0.225	29.7
3	0.1	0.380	66.8
4	0.12	0.474	90.7
5	0.16	0.600	127.7
direction of gas flow: Z			
1	0.04	0.078	7.3
2	0.08	0.247	39.1
3	0.1	0.358	61.8
4	0.12	0.452	85.3
5	0.16	0.600	128.7
average values XYZ			
1	0.04	0.071	6.3
2	0.08	0.248	36.1
3	0.1	0.371	63.9
4	0.12	0.467	87.8
5	0.16	0.600	128.3

## Data Availability

The data presented in this study are available on request from the corresponding author.

## Nomenclature

**List of major signs**		
*a*	edge of the cube	m
*b*	vector of mass forces (acceleration of gravity)	m/s^2^
*c*	constant	
*d*	diameter	m
*e*	unit base vector	
*g*	earth acceleration	m/s^2^
*k*	kinetic energy of turbulence	m/s^2^
*l*	scale of vortex	
*m*	mass	kg
*n*	equivalent number of volume *	
*o*	open	
*p*	pressure	Pa
*t*	time	s
*u*	velocity	m/s
*w*	velocity	m/s
*x*	Cartesian coordinate	
*A*	layer bed cross-section, m^2^	
*D*	outer diameter of the cell	m
*K*	coefficient of permeability	m^2^
*L*	height of porous medium	m
*P*	pressure	Pa
*Q*	stream	m^3^/s
*Re*	Reynolds number	
*T*	scale of tunnel	
*V*	volume	m^3^
*ΔP*	pressure drop, resistance flow	Pa
*Δh*	denotes pressure drop	Pa
*δ*	Kronecker symbol	
*ε*	dissipation of kinetic energy of turbulence	m^2^/s^−3^
*η*	viscosity	Pa·s
*λ*	the Kolmogorov scale	
*ρ*	density	kg/m^3^
*τ*	stress	Pa
*ψ*	indicator porosity	
*ω*	vortex	
*ϑ*	density of a solid body	kg/m^3^
*Ψ*	porosity	
**Lower indices refer to**		
1, 2, 3	Cartesian components	
*b*	absolute	
*cr*	critical value	
*d*	particle diameter	
*e*	equivalent	
*g*	gas	
*k*	ball	
*o*	reference	
*poz*	apparent	
*pr*	sample	
*st*	skeleton	
*swo*	free	
*zm*	measured	
*V*	own model	
